# Predictors of post-operative outcomes in patients with peripheral arterial disease and critical limb ischaemia: a systematic review and meta-analysis

**DOI:** 10.4314/gmj.v55i1.10

**Published:** 2021-03

**Authors:** Lily P Wu, Nadraj G Naidoo, Olatunji O Adetokunboh

**Affiliations:** 1 University of Ghana College of Health Sciences, Surgery Department, Legon, Accra, Ghana; 3 South African Medical Research Council South African Medical Research Council, Tygerberg, South Africa; 4 University of Cape Town Faculty of Health Sciences, Surgery, Observatory, Western Cape, South Africa

**Keywords:** Peripheral arterial disease, critical limb ischemia, post-operative outcomes

## Abstract

**Background:**

A very small proportion (1%) of patients with peripheral artery disease (PAD) present with critical limb threatening ischaemia (CLTI) with poor prognosis. The present review showcased several pre-operative predictors and key post-operative outcomes. Identification of any modifiable predictors may impact positively on surgical outcomes.

**Design:**

PubMed/Medline, Google scholar and Cochrane databases were searched using terms such as “peripheral arterial disease” AND “critical limb ischemia,” “post-operative outcome,” AND “predictors of post-operative outcomes”. Search was for relevant English-language articles published between January 1997 and December 2007 Selected articles were screened first by title and abstract, and selection of full articles was based on relevance using our inclusion and exclusion criteria and quality ratings performed with the MINORS score.

**Results:**

The included studies were published between 1997 and 2007. Only six (6) articles out of a total of 2,114 were deemed suitable for analysis. Ambulatory recovery was >70% at six months, 86.7% and 70.0% at one year and five years respectively. Rate of local wound complications was between 12% and 24%. Reported limb salvage rates were >90% at six months, >70% at one year and 70.0–90.0% at five years. Primary graft patency rate at one year ranged from 63% and 76.6%. Gangrene, diabetes and impaired pre-operative ambulatory function are associated with more wound complications, low limb salvage, reduced graft patency and poor functional outcome.

**Conclusion:**

Pre-operative ambulatory status was the most important predictor of post-operative ambulatory recovery. Diabetes mellitus was an important risk factor for prolonged wound healing, local wound complications and major amputation.

**Funding:**

None declared

## Introduction

Assessment of post-operative outcomes of vascular intervention for CLTI is increasingly shifting from traditional technical outcomes such as graft patency and limb-salvage,^1^ to functional outcomes such as ambulatory recovery and wound healing rates.^2^–^6^ Functional outcomes have been shown in recent reports to be better outcome measures to assess clinical success of operative interventions for CLTI. Simons et al. showed that despite adequate (>70%) graft patency and limb salvage, 10% of patients with patent grafts at 1year still could not attain clinical success of ambulatory recovery and independent living.^5^ In a study by Taylor et al, clinical success was defined as achieving all the following criteria: graft patency to the point of wound healing; limb salvage at one year; maintenance of ambulatory status for one year; and survival at six months.

Despite > 70% limb salvage and graft patency at three years, only 44.4% of the patients achieved the composite endpoint of clinical success as stated above.^3^ Goshima et al showed that time to heal exceeded three months in >50% of patients and that diabetes mellitus was a risk factor for prolong wound healing.^2^ Taylor et al. have suggested that a combination of traditional and functional outcomes is superior in the assessment of post-operative outcome post-intervention for CLTI.^3^

We undertook this meta-analysis in an effort to show-case-from the available literature – what specific pre-operative predictors most definitely affect both technical and functional post-operative outcomes.

## Methods

### Literature search

PubMed/Medline, Google scholar and Cochrane databases were systematically searched for relevant Englishlanguage articles published between January 1997 and December 2007 using the terms “peripheral arterial disease”, “critical limb ischemia,” AND “predictors of postoperative outcome”. Selected articles were screened first by title and abstract, and selection of full-text articles was based on relevance. This study was approved by the Departmental (Surgical) Research Committee of Groote Schuur Hospital and the Human Research Ethics Committee of the University of Cape Town.

Inclusion criteria was published articles on CLTI involving patients' risk factor profile and at least three post-operative outcome measures (technical, functional or both). Reviews, case reports, abstracts only, meta-analysis and non-English articles and duplicate articles were excluded. Primary endpoint was technical and/or functional post-operative outcomes. Secondary endpoint was predictors of post-operative outcomes.

### Validity assessment

**Figure 1:**
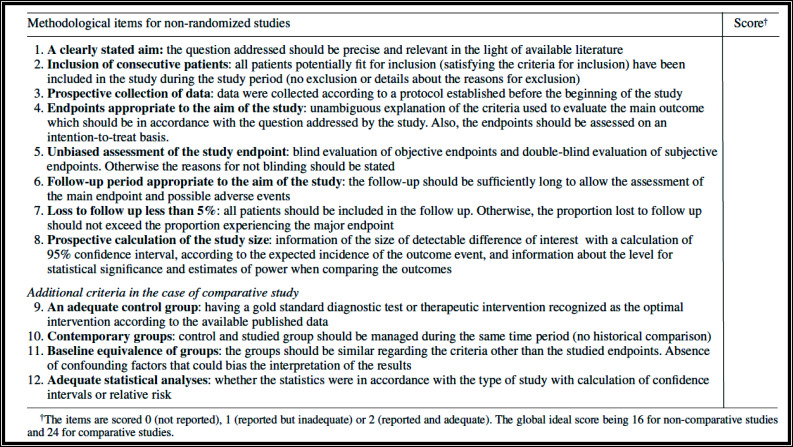
MINORS quality score

Article screening, selection and data extraction were done by two individuals independently to minimize bias. Identified relevant titles were selected as an initial step. All abstracts were read, and articles with clearly stated objectives of evaluation of predictors of post-operative outcomes of surgical treatment of CLTI were chosen. Full articles were read, and selection made based on the presence of at least three outcome measures as defined in the reporting standards of revascularization for CLTI (technical outcome measures) and/or functional outcomes or both.^7^ All related publications/electronic links of the selected articles were downloaded and the references carefully reviewed to generate any further relevant publications. The quality of the selected articles was assessed using the MINORS quality score, with a maximum score of 16 for non-comparative studies and 24 for comparative studies (Figure 1).^8^

## Results

### Description of studies

The literature search yielded 2,114 publications. A total of 1,147articles were selected after excluding irrelevant articles based on the titles and abstracts.

**Figure 2:**
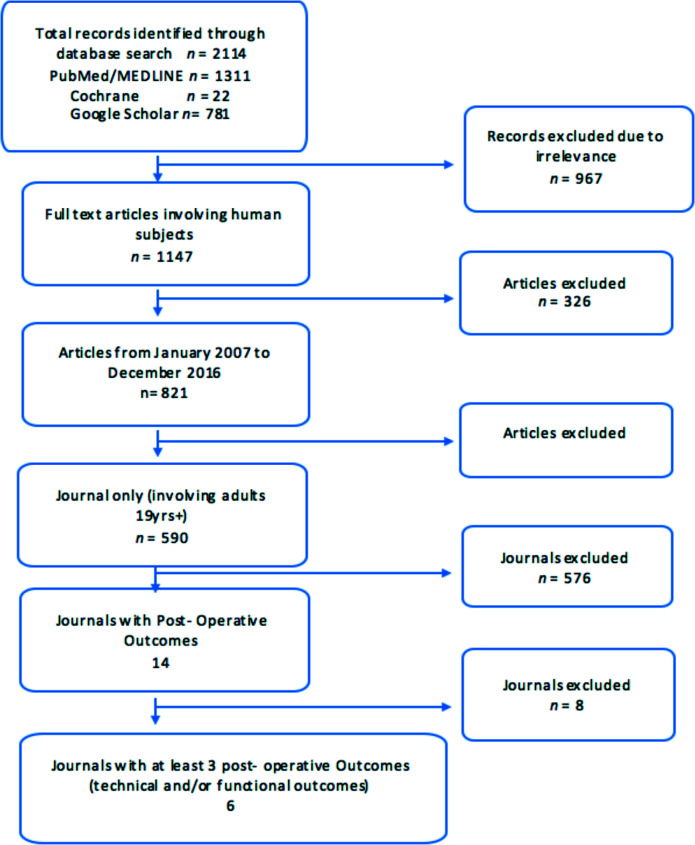
Flowchart of literature Search

Over a 10-year period, 821 articles were selected. Based on human subjects above the age of 19 years, 590 articles were identified. Only six publications were identified as suitable for our study with three or more post-operative outcome measures (Figure 2). The included studies were published between 1997 and 2007. MINORS scoring scale (Table 1).

**Table 1:** MINORS Score (6 Best performing Studies)

MINORS Score	Study Number (see KEY)					
	1	2	3	4	5	6
**A clearly stated aim**	2	2	2	2	2	2
**Inclusion of consecutive patients**	2	2	2	2	2	2
**Prospective collection of data**	0	0	2	0	0	0
**Endpoints appropriate to the aim of the** **study**	2	2	2	2	2	2
**Unbiased assessment of the study endpoints**	2	2	2	2	2	2
**Follow-up period appropriate to the aim of** **the study**	1	2	2	0	2	2
**Loss to follow up less than 5%**	2	0	2	0	0	0
**Prospective calculation of the study size**	0	0	0	0	0	0
**Total**	11	10	14	8	10	10

KEY: 1. Goshima et al, 2. Taylor et al, 3. Taylor et al 4. Abou-Zamzam et al, 5. Nicoloff et al, 6. Chung et al.

The selected studies were of moderate quality using the MINORS scoring scale (Table 1).

The total sample size of the six eligible studies was 2,446 (Sample size range: 112–841patients) with a mean follow-up of 40.5 months for four studies. In the remaining two studies, the duration of follow up was not stated. All six studies reported a combination of technical and functional outcomes but at different timelines. Technical outcome measures were based on the standards for reporting by the Society for Vascular Surgery/International Society for Cardiovascular Surgery.^1^,^7^ Five of them were retrospective studies and one was a prospective study (Table 2a). The study population was grouped based on the investigated outcome measures and timelines at which the outcomes were assessed (Table 2b).

**Table 2a:** Included studies

Study	n	Type of Study	Follow up (months)	Type of operation
**Goshima et al 2004** 2	315	Retrospective	Not reported	Infra-inguinal bypass surgery
**Taylor et al 2007** 3	331	Retrospective	72	Infra-inguinal and supra-inguinal bypass surgery
**Taylor et al 2006** 4	841	Prospective	60	Open revascularization, Endovascular intervention and hybrid procedures
**Abou-Zamzam et al 1997** 9	513	Retrospective	Not reported	Infra-inguinal bypass surgery
**Nicoloff et al 1998** 6	112	Retrospective	36	Infra-inguinal bypass surgery
**Chung et al 2006** 10	334	Retrospective	30	Infra-inguinal bypass surgery
**Total**	2,446			

n = Number of Patients (Sample size)

**Table 2b:** Included studies

Study		Goshima et al 2004	Taylor et al 2007	Taylor et al 2006	Abou-Zamzam et al 1997	Nicoloff et al 1998	Chung et al 2006
**Limb Salvage (%)**	6 months				94.2	94.9	85
	1 year		80.6	76.5			
	3 years					93.5	79.0
	5 years	91		72.1	85.2		
**Primary Graft Patency** **(%)**	6 months				92.0	93.1	
	1 year			76.6			63.0
	3 years					87.6	50.0
	5 years			72.4			
**Ambulatory recovery** **(%)**	6 months				80.0	92.0	72.0
	1 year		86.7				
	3 years					73.0	
	5 years			70.6			
**Wound Healing (%)**	6 months	54.0			12.0		42.0
	1 year						75.0

### Outcome measures and predictive factors

Ambulatory recovery varied significantly, 72% to 92% at six months, 86.7% at one year, 73% at three years and 70.6% at five years. Rates of local wound complications reported were between 12% and 24%. In some studies, more than 50% of the ischemic wounds took longer than four months to heal. In one study, the wound healing rates at six months and one year were 42% and 75% respectively. Reported limb salvage rates were from 94.2% at six months to 76.5–85.0% at one year. The five years' limb salvage rates were 72.1–91%. Primary graft patency rate at one year was between 63% and 76.6%. Major amputation rates varied widely from 3.5% at three months to 23.2% at three years. Survival at six months, one year and five years were >80%, 89% and 48% respectively.

Table 2c shows some of the reported pre-operative risk factors. Only three included studies expressed those factors as odds ratios or hazard ratios while others reported as percentage or just in narrative format. Multiple factors influenced post-operative outcomes. Tissue loss and diabetes mellitus were associated with multiple re-operations and prolong wound healing.^2^,^10^ Gangrene, Diabetes, ESRD and impaired pre-operative ambulatory function predicted low limb salvage, reduced graft patency and poor functional outcome.^4^,^9^ Pre-operative impaired ambulatory function was reported in most of the studies to be the most important predictor of sub-optimal post-operative functional outcome.^4^,^9^,^11^ A composite endpoint of limb salvage and ambulation at one year, survival at six months and graft patency up to the point of healing was achieved in only 44% of patients.^3^

**Table 2c:** Included studies with reported pre-operative risk factors

Included studies	Outcomes	Reported pre-operative risk factors
**Goshima [2]**	Delayed wound healing	Diabetes mellitus (odds ratio [OR] = 3.4)
	Reoperation < 3 months	Ischemic tissue loss (OR = 3.1)
		Minority status (OR = 2.2)
	Readmission < 6 months	Ischemic tissue loss (OR = 2.8)
		Renal failure (OR = 2.3)
**Taylor [3]**	Impaired ambulatory ability at the time of presentation	5-year mortality (hazard ratio [HR] = 3.34)
		Failure to eventually ambulate (HR = 2.83)
		Loss of independent living status (HR = 7.97)
	The presence of dementia	Late mortality (HR = 1.57)
		Failure to eventually ambulate (HR = 2.20)
		Loss of independent living status (HR = 5.44)
**Taylor [4]**	Surgical revascularization failure	Impaired ambulatory status at presentation (OR = 6.44)
		Presence of infrainguinal disease (OR = 3.93)
		End-stage renal disease (OR = 2.48)
		Presence of gangrene (OR = 2.40)
		Hyperlipidemia (OR = 0.56)

Some studies investigated predictors of post-operative outcome but the spectrum of outcome measures assessed were fewer than the minimum of 3 selected for our study and therefore were excluded.^5^,^12^ Other studies with sample population of patients who had re- intervention for failed previous vascular interventions were also excluded (Table 3).^13^,^14^

**Table 3:** Excluded studies n = Number of Patients (Sample size)

Source	Type of study	n	Reasons for exclusion	Conclusion
**Kudo et al**	Retrospective	192	Different objective (procedure volume over different time-period and outcomes)	open revascularization for the treatment of CLI have been largely replaced by angioplasty procedures without compromising outcomes
**Vinit et al**		331	Review	Patients with CLI should be offered revascularization if the procedure can be tolerated and the patient is ambulatory and living independently preoperatively. Amputation should be considered if the above criteria is not met.
**Goodney et al**	Retrospective	2,031	<3 post-operative outcomes. Examined risk factors that predict amputation or graft occlusion within the first year following lower extremity bypass.	Risk factors can predict the risk of amputation and graft occlusion post-revascularization for CLI
**Simons et al**	Retrospective	513	Less than 3 post-operative outcome measures investigated (assessed predictors of clinical failure defined as amputation and worsening ischaemia at 1 year)	10% of patients with patent grafts at 1year still could not attain clinical success. One of the predictors of failure to attain clinical success was ESRD.
**Rutherford et** **al**			Review	The initial published standards for reporting post-operative outcome for CLI requires periodic revision
**Gibbons et al**	Retrospective	318	Qualitative study	Baseline health status is a predictor of post-operative functional recovery and well-being.
**Kudo et al**	Retrospective	111	Evaluated post-operative outcome (effectiveness) of only percutaneous transluminal angioplasty (PTA)	PTA is feasible, safe and effective for the treatment of CLI
**Ambler et al**	Retrospective	90	Evaluated only 1 outcome measure, ambulation	Poor pre-operative ambulation predict poor post-operative ambulation and long LOS
**Total**		3,586		

Out of the total of six eligible studies, only five studies were used in conducting the meta-analysis. The study that was excluded from meta-analysis did not report the outcomes at the timelines of interest. There is a high level of heterogeneity in the timelines for the various outcome measures in the papers evaluated (Figure 3a, b, c, d). Multiple factors predict post-operative outcomes.

**Figure 3a:**
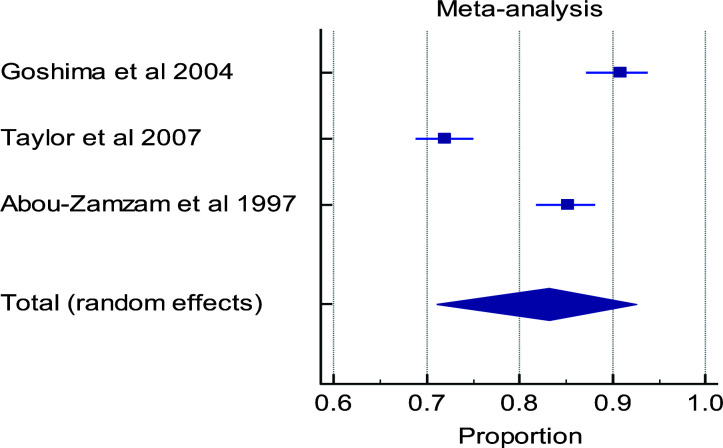
Forest plot of meta-analysis of the proportion estimates for post-operative outcome: limb salvage

**Table:** 

Study	Sample size	Proportion (%)	95% CI	Weight (%)	Test for heterogeneity	
				Random	Q	69.3607
**Goshima et al 2004**	318	90.811	87.166 to 93.807	32.82	DF	2
**Taylor et al 2007**	841	72.057	68.891 to 75.067	33.78	Significance level	P < 0.0001
**Abou-Zamzam et al** **1997**	513	85.185	81.811 to 88.147	33.40	I^2^ (inconsistency)	97.12%
**Total (random effects)**	1672	83.229	70.999 to 92.617	100.00	95% CI for I^2^	94.24 to 98.56

**Figure 3b:**
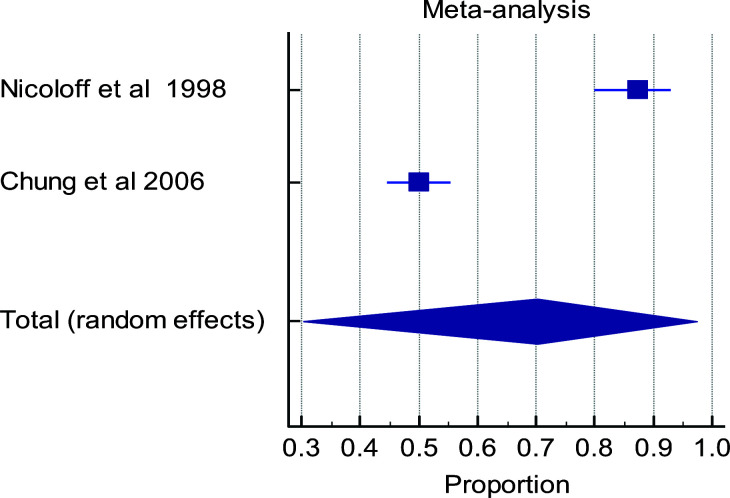
Forest plot of meta-analysis of the proportion estimates for post-operative outcome: primary graft patency

**Table:** 

Study	Sample size	Proportion (%)	95% CI	Weight (%)	Test for heterogeneity	
				Random	Q	59.3637
**Nicoloff et al 1998**	112	87.500	79.917 to 92.995	49.58	DF	1
**Chung et al 2006**	334	50.000	44.509 to 55.491	50.42	Significance level	P < 0.0001
**Total (fixed effects)**	446	60.492	55.797 to 65.048	100.00	I^2^ (inconsistency)	98.32%
**Total (random effects)**	446	70.187	30.262 to 97.240	100.00	95% CI for I^2^	96.22 to 99.25

**Figure 3c:**
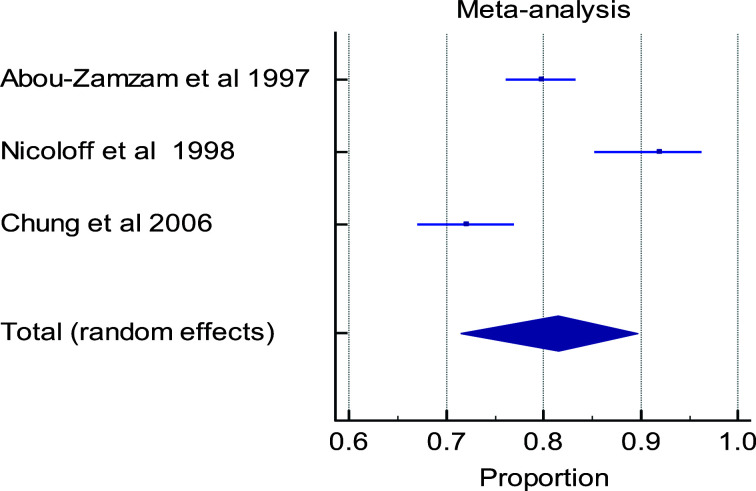
Forest plot of meta-analysis of the proportion estimates for post-operative outcome: ambulatory recovery

**Table:** 

Study	Sample size	Proportion (%)	95% CI	Weight (%)	Test for heterogeneity	
				Random	Q	23.8936
**Abou-Zamzam et al 1997**	513	79.922	76.190 to 83.305	35.31	DF	2
**Nicoloff et al 1998**	112	91.964	85.293 to 96.260	30.25	Significance level	P < 0.0001
**Chung et al 2006**	334	72.156	67.016 to 76.897	34.44	I^2^ (inconsistency)	91.63%
**Total (random effects)**	959	81.472	71.495 to 89.699	100.00	95% CI for I2	78.61 to 96.73

**Figure 3d:**
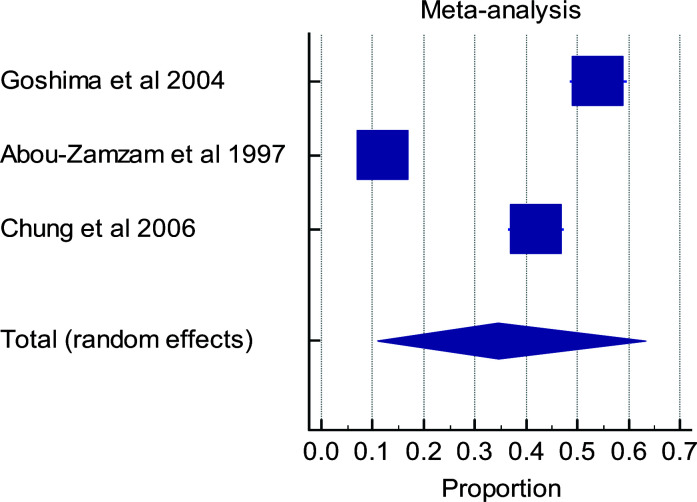
Forest plot of meta-analysis of the proportion estimates for post-operative outcome: wound healing

**Table:** 

Study	Sample size	Proportion (%)	95% CI	Weight (%)	Test for heterogeneity	
				Random	Q	200.9922
**Goshima et al 2004**	318	54.088	48.437 to 59.662	33.28	DF	2
**Abou-Zamzam et al 1997**	513	12.086	9.393 to 15.224	33.43	Significance level	P < 0.0001
**Chung et al 2006**	334	41.916	36.567 to 47.410	33.30	I^2^(inconsistency)	99.00%
**Total (random effects)**	1165	34.571	10.951 to 63.280	100.00	95% CI for I^2^	98.37 to 99.39

## Discussion

There is a high level of heterogeneity in the timelines for the various outcome measures in the papers evaluated. Multiple factors predict post-operative outcomes. Goshima et al studied the outcomes after infrainguinal bypass surgery to determine risk factors for adverse outcomes. Time to heal exceeded three months in >50% of patients and diabetes mellitus among other factors was a major risk factor for prolong wound healing.^2^ In a study by Taylor et al, predictors of failure to achieve clinical success were the presence of gangrene, ESRD, hyperlipidaemia and impaired pre-operative ambulatory function.^3^ In that same study by Taylor et al, clinical success was defined as achieving all the following criteria: graft patency to the point of wound healing; limb salvage at one year; maintenance of ambulatory status for one year; and survival at six months. Despite > 70% limb salvage and graft patency at three years, only 44.4% of the patients achieved the composite endpoint of clinical success as stated above.^3^ Taylor et al again examined the determinants of functional outcome after revascularization for critical limb ischemia. Findings were that, at five years, graft patency and limb salvage were acceptable at 70%. However, functional outcomes at five years were low, survival was only 41% with 70% ambulatory recovery.^4^ Sub-optimal post-operative ambulation was predicted by poor pre-operative ambulatory function.^4^

The large number of retrospective studies did not allow for standardization of timelines for outcome measurement. In terms of the type of revascularization procedure, many studies reported on only infra-inguinal bypass surgeries, but some studies reported also on supra-inguinal bypass as well as endovascular interventions. This makes it difficult to conduct comparative analysis. The small number of studies elligible for the meta-analysis resulted in restricted analysis and non-comprehensive results. There may be recall and selection bias due to the large number of retrospective studies.

## Conclusion

There is wide variation in the parameters and timelines for reporting post-operative outcomes. This limits the effectiveness and quality of systematic review and accounts for the heterogeneity of the selected studies for the meta-analysis. Many more patients who were ambulant pre-operative continued to ambulate post-operative than those who were non-ambulant pre-operative. Diabetes mellitus was an important risk factor for prolonged wound healing, local wound complications and major amputation. Effective pre-operative glycemic control may improve post-operative outcome.
